# Evaluation of 12-lead electrocardiogram at 0.55T for improved cardiac monitoring in magnetic resonance imaging

**DOI:** 10.1016/j.jocmr.2024.101009

**Published:** 2024-02-10

**Authors:** Aravindan Kolandaivelu, Christopher G. Bruce, Felicia Seemann, Dursun Korel Yildirim, Adrienne E. Campbell-Washburn, Robert J. Lederman, Daniel A. Herzka

**Affiliations:** a Cardiovascular Branch, Division of Intramural Research, National Heart Lung and Blood Institute, National Institutes of Health, Bethesda, Maryland, USA; b Division of Cardiology, Department of Medicine, Johns Hopkins University School of Medicine, Baltimore, Maryland, USA; c Department of Radiology, Case Western Reserve University and University Hospitals Cleveland Medical Center, Cleveland, Ohio, USA

**Keywords:** Low-field MRI, Electrocardiogram, Arrhythmia, Myocardial ischemia, Magnetohydrodynamic effect

## Abstract

**Background:**

The 12-lead electrocardiogram (ECG) is a standard diagnostic tool for monitoring cardiac ischemia and heart rhythm during cardiac interventional procedures and stress testing. These procedures can benefit from magnetic resonance imaging (MRI) information; however, the MRI scanner magnetic field leads to ECG distortion that limits ECG interpretation. This study evaluated the potential for improved ECG interpretation in a “low field” 0.55T MRI scanner.

**Methods:**

The 12-lead ECGs were recorded inside 0.55T, 1.5T, and 3T MRI scanners, as well as at scanner table “home” position in the fringe field and outside the scanner room (seven pigs). To assess interpretation of ischemic ECG changes in a 0.55T MRI scanner, ECGs were recorded before and after coronary artery occlusion (seven pigs). ECGs was also recorded for five healthy human volunteers in the 0.55T scanner. ECG error and variation were assessed over 2-minute recordings for ECG features relevant to clinical interpretation: the PR interval, QRS interval, J point, and ST segment.

**Results:**

ECG error was lower at 0.55T compared to higher field scanners. Only at 0.55T table home position, did the error approach the guideline recommended 0.025 mV ceiling for ECG distortion (median 0.03 mV). At scanner isocenter, only in the 0.55T scanner did J point error fall within the 0.1 mV threshold for detecting myocardial ischemia (median 0.03 mV in pigs and 0.06 mV in healthy volunteers). Correlation of J point deviation inside versus outside the 0.55T scanner following coronary artery occlusion was excellent at scanner table home position (r^2^ = 0.97), and strong at scanner isocenter (r^2^ = 0.92).

**Conclusion:**

ECG distortion is improved in 0.55T compared to 1.5T and 3T MRI scanners. At scanner home position, ECG distortion at 0.55T is low enough that clinical interpretation appears feasible without need for more cumbersome patient repositioning. At 0.55T scanner isocenter, ST segment changes during coronary artery occlusion appear detectable but distortion is enough to obscure subtle ST segment changes that could be clinically relevant. Reduced ECG distortion in 0.55T scanners may simplify the problem of suppressing residual distortion by ECG cable positioning, averaging, and filtering and could reduce current restrictions on ECG monitoring during interventional MRI procedures.

## Graphical abstract

**Figure:**
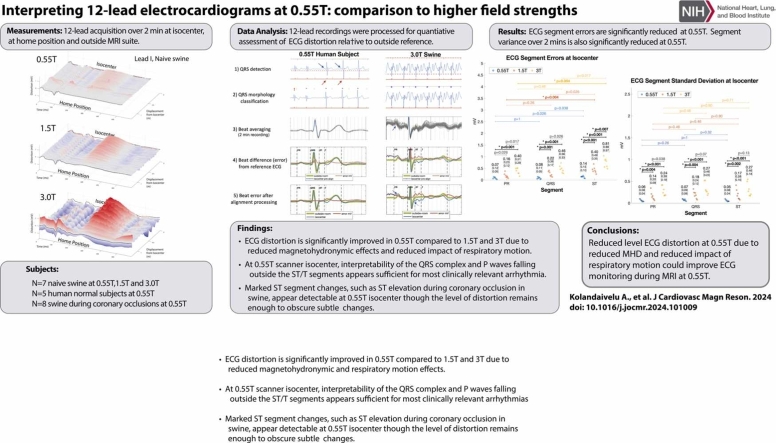
This study evaluated ECG interpretability in a 0.55T MRI scanner. The workflow for comparing 0.55T to higher-field MRI is shown. ECG distortion improved in 0.55T compared to 1.5T and 3T MRI scanners. At scanner home position, ECG distortion at 0.55T was low enough that clinical interpretation appears feasible without need for more cumbersome patient repositioning. At 0.55T scanner isocenter, ST segment changes during coronary artery occlusion appear detectable but distortion was enough to obscure subtle ST segment changes that could be clinically relevant. ECG: electrocardiogram, MRI: magnetic resonance imaging.

## 1 Background

The 12-lead electrocardiogram (ECG) is a standard diagnostic tool for monitoring cardiac ischemia and heart rhythm during cardiac interventional procedures and stress testing. There is interest in performing these procedures inside the magnetic resonance imaging (MRI) scanner to incorporate MRI information, such as myocardial perfusion, blood flow, and ablation lesion characterization [1], [2], [3]. However, the ECG interpretation typically required during these procedures is limited in the strong magnetic field of the MRI scanner. Blood flowing perpendicular to the MRI static magnetic field, in particular the aortic arch, generates a voltage due to separation of charged ions in the blood from the Hall effect [4], [5]. This induced voltage is also termed the magnetohydrodynamic effect (MHD) and is superimposed on the surface ECG. MHD voltage is highest during ventricular systole which follows the ECG QRS complex. This significantly obscures the ST segment, which is important for assessment of cardiac ischemia [6], [7].

Jekic et al. identified a magnetic field threshold of below 70 mT at which ECG interpretation was accurate [8]. This could be achieved outside a 1.5T MRI scanner when subjects were positioned feet-first into the scanner. However, this positioning limits use for monitoring during cardiac procedures and is inconvenient for exercise stress testing where image acquisition must be completed within 60 s of stopping exercise and ECG monitoring is continued after imaging [9].

Since MHD decreases with lower magnetic field strength, one benefit of performing interventional procedures in low field MRI could be improved ECG interpretability. Another benefit of low field MRI for interventional procedures is the reduced risk of device heating [10]. Since the specific absorption rate (SAR) is proportional to the square of magnetic field strength, low field (0.55T) imaging reduces heating around sevenfold relative to 1.5T and 30-fold relative to 3T. With lower field strength, additional existing devices can be safely deployed during intervention without risk of heating and design and fabrication of new devices is simplified [11], [12]. The commercial availability of low field scanners is additionally driven by reduced installation and maintenance cost and reduced sensitivity to magnetic susceptibility artifacts [11], [13]. The latter has potential advantages for pulmonary imaging, imaging around magnetic implants, and abdominal imaging. The potential benefits of low field MRI need to be weighed against the reduction in signal-to-noise ratio (SNR) compared to higher field MRI, which impacts achievable image resolution and scan duration.

In this study, we evaluated 12-lead ECG fidelity for a low field 0.55T MRI scanner compared to 1.5T and 3T scanners in a porcine model. Assessment of ischemic ECG changes in the 0.55T scanner was performed before and after coronary artery occlusion. Human volunteer ECG interpretation at 0.55T was also evaluated at rest and following ergometer exercise.

## 2 Methods

Animal protocols were reviewed and approved by the Animal Care and Use Committee at the National Institutes of Health. The healthy volunteer protocol was approved by the Institutional Review Board of the National Institutes of Health (ClinicalTrails.gov identifier NCT03331380), and all subjects provided written informed consent.

### 2.1 12-Lead ECG recording protocol

An MRI conditional ECG system was used for recording 12-lead ECGs with 1000 Hz sampling frequency (PinMed, Pittsburgh, Pennsylvania, USA). The 10 ECG electrodes with carbon fiber wires were positioned in standard fashion. A reference 12-lead ECG was first acquired outside the scanner room. With the subject headfirst in the MRI scanner and the scanner table at “home” position, electrodes V1 and V2 were confirmed to be at 60 cm from the landmark point at the entrance of the scanner bore for positioning consistency. ECG was then recorded at the home position and recorded again after advancing electrodes V1 and V2 to isocenter. Recordings were taken for 2 min at each location to assess beat-to-beat variation in ECG morphology and compare average beat morphology. In a subset of subjects, a repeat ECG was recorded outside the scanner room to assess variation of the reference ECG.

### 2.2 12-Lead ECG comparison at 0.55T, 1.5T, and 3T in animals and 0.55T in human subjects

Yorkshire swine, 40–50 kg, (N = 7) were studied under general anesthesia with isoflurane (1–2%) and mechanical ventilation. The 12-lead ECG recording protocol was performed in a 0.55T MRI scanner (prototype MAGENTOM Aera, Siemens, Erlangen, Germany). Animals were then transported, and ECG recording protocol repeated in a 1.5T MRI scanner (MAGENTOM Aera, Siemens, Erlangen, Germany) and a 3T MRI scanner (Biograph mMR, Siemens, Erlangen, Germany). The 12-lead ECG recording protocol was also performed in five healthy volunteers in the 0.55T MRI scanner.

### 2.3 12-Lead ECG assessment at 0.55T after coronary occlusion in animals

Yorkshire swine, 40–50 kg, (N = 7) were studied under general anesthesia with isoflurane (1–2%) and mechanical ventilation. The 12-lead ECG recording protocol was first performed in the 0.55T MRI scanner prior to coronary occlusion. In a fluoroscopy suite adjacent to the MRI scanner, x-ray guidance was used to advance a coronary guide catheter into the aortic root via femoral arterial access. Percutaneous coronary intervention angioplasty balloon inflation was performed within the target coronary vessel and occlusion confirmed by angiography. Balloon inflation was maintained after disconnecting the inflator device using a stopcock. After 10–15 min, ST segment deviation reached a qualitatively stable appearance. The 12-lead ECG recording protocol was then repeated during ongoing coronary balloon occlusion.

### 2.4 Changes in ST segment distortion with ergometer stress at 0.55T in human subjects

An MRI-compatible pedal ergometer (Cardio Step Module, Ergospect, Innsbruck, Austria) was used to evaluate stress related changes in the ST segment in the 0.55T scanner. In five additional healthy volunteers, continuous 12-lead ECG was recorded at rest prior to exercise, during ergometer exercise, and during exercise recovery after pedaling was stopped. Pedal resistance was increased in a stepwise manner every 30 s to 60 s until the subject felt unable to continue exercise. This stress protocol was performed first at home position. After return of the heart rate toward the resting heart rate, the stress protocol was repeated after advancing ECG electrodes V1 and V2 to isocenter.

### 2.5 Relationship of aortic flow to ST segment distortion at 0.55T

In 5 healthy volunteers, 12-lead ECG was recorded for 2 min first with the scanner table at home position and then after advancing electrodes V1 and V2 to isocenter. Immediately after ECG recording at isocenter, phase contrast imaging of flow through the proximal ascending aorta was performed using standard chest and spine array coils (30 frames/heartbeat, velocity encoding (VENC) set to 200 cm/s, TE/TR 4.32/28.12 ms, 192 × 144 matrix, field of view (FOV) 360 × 270, slice 6 mm, bandwidth 300 Hz/pixel, 3 averages).

### 2.6 Data analysis

ECG signal processing and statistical analysis were performed using MATLAB (MathWorks, Natick, Massachusetts). The ECG for sinus rhythm heartbeats was obtained as follows. ECG recordings were bandpass filtered, 0.67–150 Hz, consistent with guideline recommendations [14].

**Fig. 1:**
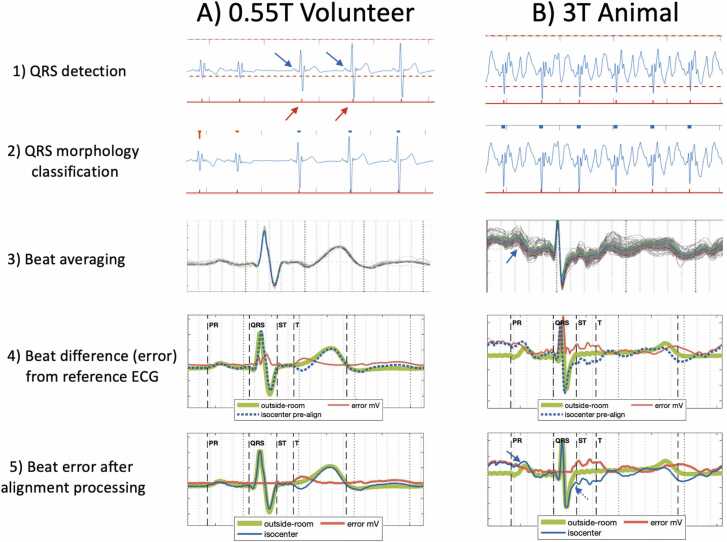
ECG processing methodology. Column (A) illustrates processing steps for a healthy human volunteer ECG recorded at 0.55T isocenter and column (B) illustrates the processing steps for a porcine subject ECG recorded at 3T isocenter. Step (1) A threshold was manually adjusted (dashed red line) to pass through QRS complexes while excluding the T wave. Detected QRS complexes are marked below the ECG tracing (red arrows and tick marks). Blue arrows highlight that at 0.55T low amplitude features, such as P waves, can be discerned before additional processing steps. Step (2) Examples of each different QRS morphology in the ECG recording are manually selected as QRS “templates.” In the 0.55T example, the smaller amplitude QRS complex was selected as one template and the larger amplitude QRS complex was selected as another template. The remaining QRS complexes are assigned to the template with the greatest cross-correlation. Step (3) Heartbeats matching the most frequent “normal sinus beat” QRS template are averaged after alignment of the QRS complexes to maximize cross-correlation. The ECGs used for averaging are plotted in gray and the resulting averaged ECG plotted in blue. One standard deviation bounds are show in green and red. The blue arrow illustrates that at 3T low amplitude features, such as the P wave, can fall within beat-to-beat ECG variation making detection more difficult. Steps (4) and (5) illustrate the methodology for evaluating error (red tracing) of the ECG tracing at isocenter (blue tracing) relative to the ECG outside the scanner room (green tracing). The PR, QRS, and ST, and T intervals are manually marked to assess error in different portions of the ECG. Step (4) shows simple subtraction results in overcalled “error” during the QRS complex that does not correspond to clinically significant differences in QRS morphology. Step (5) illustrates the effect of the alignment processing described in the Methods for suppressing sub-clinical QRS error while preserving error in other portions of the ECG. The solid blue arrow highlights at 3T low amplitude features, such as the P wave, can be obscured by ECG error. The dashed blue arrow highlights the shape of the higher amplitude QRS complex is better preserved at 3T, but error can be seen in later portions of the QRS complex. ECG: electrocardiogram.

The methodology for ECG processing is described below and illustrated in Fig. 1. The filtered ECG was first divided into individual heartbeats. QRS complex locations were determined by a manually set threshold below the QRS complex peak and above the T wave peak. A measured PR interval and average QRS to QRS interval (R-R interval) were used to define the heart-beat window for each QRS complex. Sinus rhythm heart beats were separated from premature ventricular complexes (PVCs) by manual selection of representative QRS “templates” for the sinus rhythm and PVC beats. All heartbeats were then assigned to a QRS template based on maximum cross-correlation. Further evaluation was restricted to heartbeats assigned to the sinus rhythm QRS template.

For quantitative assessment, the ECG for each sinus rhythm beat was first aligned to maximize cross-correlation of QRS complexes. The average and standard deviation for each sample of the aligned heartbeats were then taken. For each of the 12 leads, ECG error at scanner isocenter and home position was calculated as the maximum difference from the averaged ECG outside the scanner room. To minimize clinically relevant ECG error, the maximum difference was taken after the following additional alignment of the averaged ECG waveforms. Vertical alignment was performed to minimize the total absolute difference from the start of the P wave to the first 1/3 of the QRS complex. The remainder of the ECG was not used for vertical alignment to reduce the influence of MHD effect, which were observed during latter portions of the QRS complex, particularly in the 3T scanner. The QRS complex peak was then truncated by 5% to reduce QRS error that arose from small variations of QRS complex amplitude. This truncation is in line with the guideline suggested noise tolerance of 5% for visual ECG interpretation [14]. Lastly, fine time alignment was performed by taking the minimum difference of each point within ±5 ms. This reduced error from slight timing variations that fell below the threshold for visual interpretation (0.25 mm on standard scale ECG) [14] and would otherwise contribute to error during rapidly changing portions of the ECG, such as the QRS complex.

To evaluate the effect of ECG error and standard deviation on ECG interpretation, the following clinically relevant intervals were identified on the averaged heartbeat 12-lead ECG recorded outside the scanner room (Fig. 1, Step 4) [15], [16]. The QRS interval contains the “QRS complex” which reflects the depolarization of the ventricular myocardial transmembrane potential which precedes ventricular systole. QRS complex shape helps to rapidly distinguish potentially higher risk “wide-QRS complex” tachycardias such as ventricular tachycardia from lower risk “narrow-QRS complex” heart rhythms that originate from the atrium. The PR interval contains the “P wave” which marks the timing of atrial depolarization relative to ventricular depolarization. P wave timing is useful for distinguishing rhythms that originate in the atrium, such as distinguishing atrial tachycardia from other atrial arrhythmias. The “T wave” reflects ventricular repolarization to its baseline state in preparation for the next heartbeat. The PR and QRS intervals and end of the T wave were manually marked on the ECG by a cardiologist. ECG changes between the end of the QRS complex and T wave (termed the ST interval or ST segment) are used to diagnose myocardial ischemia and infarction. The ST segment was taken to be the end of the QRS complex (J point) to 80 ms following the J point, reflecting the interval used for assessing ischemic ST segment changes [9]. ECG error and standard deviation was determined for the entire heartbeat, the PR interval, the QRS interval, the ST segment, and at the J point for each subject and MRI scanner field strength. For each subject, the ECG lead with the maximum error was used for statistical assessment of ECG errors. The lead with the maximum standard deviation was used for statistical assessment of standard deviation. Median and inter-quartile range of ECG error and standard deviation were reported across subjects. ECG errors and standard deviations were compared by Wilcoxon rank sum test. A p-value ≤0.05 was considered significant, adjusted using Bonferroni correction accounting for the number of simultaneous comparisons [17].

Shift of the J point compared to the ECG baseline between heartbeats (ST deviation) is the primary indicator of myocardial ischemia and infarction [18]. ST deviation was determined from the averaged heartbeat 12-lead ECG during coronary occlusion. ST deviations measured at the MRI scanner home position and isocenter were compared to the ST deviation outside the scanner room by Bland-Altman analysis. Diagnostic assessment of the 12-lead ECGs for presence or absence of ischemic ST-segment changes was also performed by a cardiologist. ECGs were presented in random order to reduce bias. J point deviations of 0.1 mV or greater in at least two anatomically contiguous ECG leads was considered indicative of ischemia.

Error between individual leads and anatomically contiguous lead groups was evaluated. The anatomic lead groups were defined as follows: lateral leads I, aVL, V5, and V6, inferior leads II, III, and aVF, septal leads V1 and V2, and anterior leads V3 and V4 [20]. The lower error for each pair of anatomically contiguous leads was taken to represent each lead pair. This is consistent with the clinical interpretation of significant ST deviations as occurring in at least two anatomically contiguous leads [21], [22]. The lead pair with the maximum error was then chosen to represent each anatomic lead group.

Analysis of the healthy volunteer ergometer exercise and aortic flow imaging studies was performed as follows. For the ergometer studies, ST segment distortion of the averaged 12-lead ECG was qualitatively compared between rest and stress both at scanner table home position and at isocenter. Exercise motion-related ECG noise limited ECG interpretation both at scanner table home position and isocenter. The “stress ECG” was taken immediately after the subject stopped exercise. The 10 s intervals were used for averaging to minimize heart rate reduction after stopping exercise. For consistency, 10 s averaging was also used for the resting ECG. For the aortic flow imaging studies, the timing of peak aortic flow was compared to timing of peak ECG distortion. ECG distortion taken to be the difference between the averaged 12-lead ECG at isocenter and scanner table home position. The aortic flow from phase contrast MRI was time aligned to the ECG by setting the time of the first flow measurement to the estimated QRS trigger timing for the ECG gated phase contrast images, 10 ms before the QRS peak on the averaged ECG [19].

## 3 Results

### 3.1 Comparison of ECG distortion for 0.55T, 1.5T, and 3T MRI scanners

**Fig. 2:**
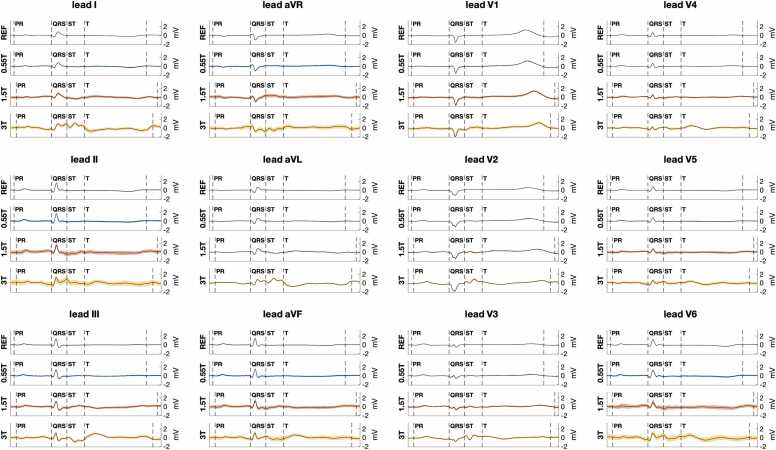
Illustration of 12-lead ECG distortion at different MRI scanner field strengths. For reference, the PR, QRS, ST, and T wave intervals are labeled for each lead. The top trace for each lead is the baseline ECG outside the scanner room (labeled REF). ECGs at MRI scanner isocenter are shown for 0.55T (second trace), 1.5T (third trace), and 3T (fourth trace). The black line for each trace is the average ECG over a 2-minute ECG recording. The colored bounds indicate the one standard deviation bound for each ECG time point. Increased ST segment and T wave distortion are seen at increasing scanner field strength. At 3T, distortion can also be seen toward the end of the QRS complex. Standard deviation bounds are wider at higher field strength but relatively uniform across different ECG intervals. All ECGs are from the same porcine subject with the same ECG lead positions. ECG: electrocardiogram, MRI: magnetic resonance imaging.

**Fig. 3:**
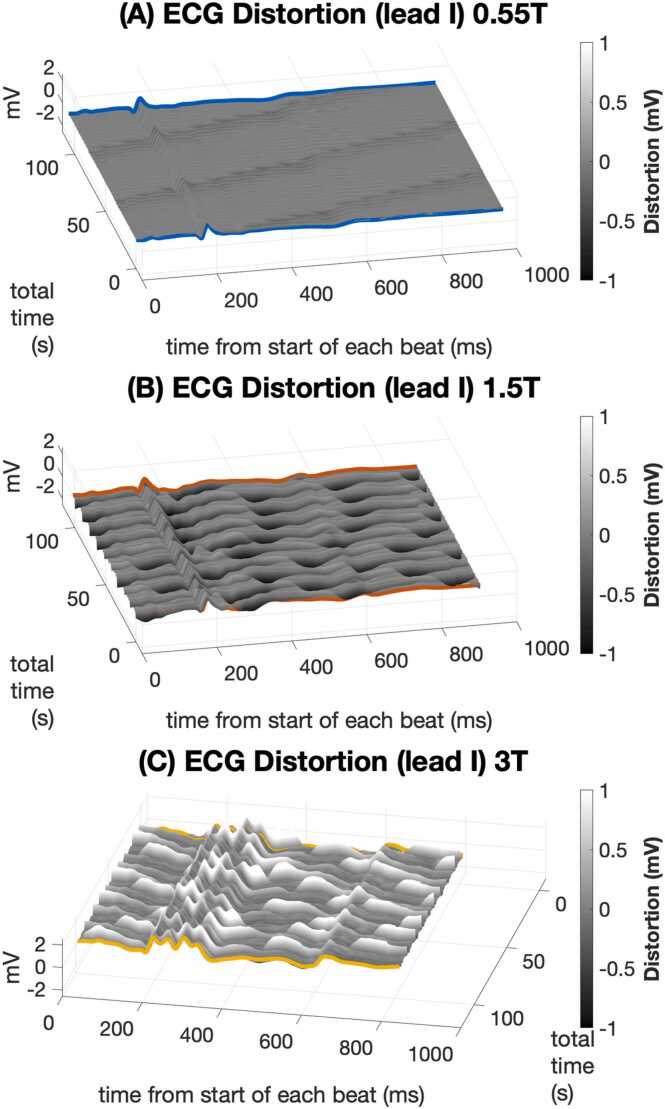
Illustration of beat-to-beat ECG variation over 2-minute recordings at different MRI scanner field strengths. The gray color scale indicates the difference of each heartbeat ECG from the 2-minute averaged ECG. Periodic variation in ECG distortion is most prominent at the respiration rate. Variation is least noticeable at 0.55T (top plot), and more conspicuous at 1.5T (middle plot), and 3T (bottom plot). ECGs are from the same porcine subject as Fig. 2 with the same ECG lead position. ECG: electrocardiogram, MRI: magnetic resonance imaging.

Fig. 2 illustrates the increased ECG distortion observed with increasing MRI scanner field strength. Distortion of the averaged heart-beat ECG occurred mostly after the start of the QRS complex, consistent with MHD effects. Beat-to-beat variation over 2-minute recordings was more uniformly distributed across the PR, QRS, and ST segments of the ECG. Fig. 3 shows that in anesthetized animals this variation was largely periodic and occurred at the mechanical ventilation rate, consistent with a respiration motion source.

**Fig. 4:**
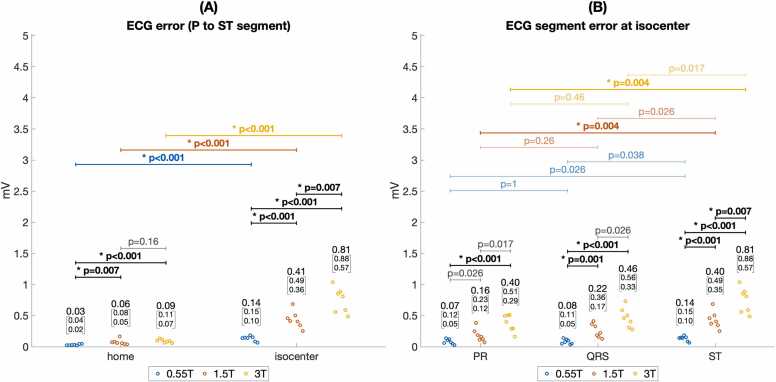
ECG error in 0.55T, 1.5T, and 3T MRI scanners. (A) ECG error increased at each field strength when moving from scanner home position to isocenter. ECG error was lower at 0.55T compared to 1.5T and 3T both at scanner isocenter and at home position. (B) Compares ECG error between different ECG intervals at scanner isocenter. At 1.5T and 3T, ST segment error was higher than P wave error. Increasing ECG error with increasing scanner field strength reached more consistent statistical significance for the ST segment than the PR and QRS intervals. These findings are consistent with maximum MHD effect occurring during the ST segment. Figure data are from porcine subjects. ECG: electrocardiogram, MRI: magnetic resonance imaging.

**Fig. 5:**
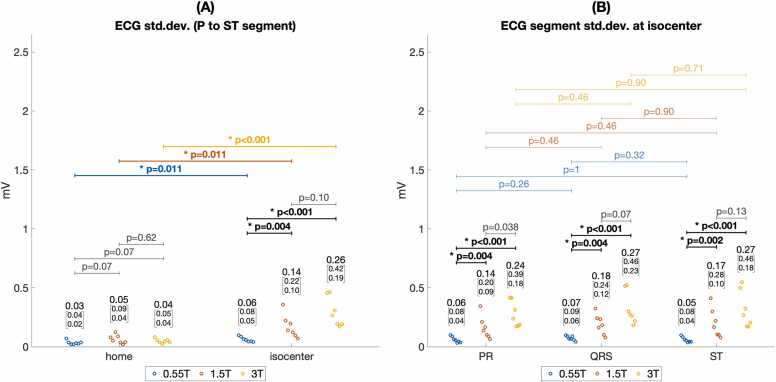
ECG variation in 0.55T, 1.5T, and 3T MRI scanners. (A) Shows ECG variation increased at each field strength when moving from scanner home position to isocenter. At isocenter, ECG variation was significantly lower for 0.55T compared to 1.5T and 3T scanners. (B) Compares ECG variation between different ECG intervals at scanner isocenter. ECG variation was significantly lower at 0.55T compared to 1.5T and 3T for all intervals. However, in contrast to ECG error (Fig. 4), ECG variation was not significantly different between the PR, QRS, and ST intervals. Figure data are from porcine subjects. ECG: electrocardiogram, MRI: magnetic resonance imaging.

**Fig. 6:**
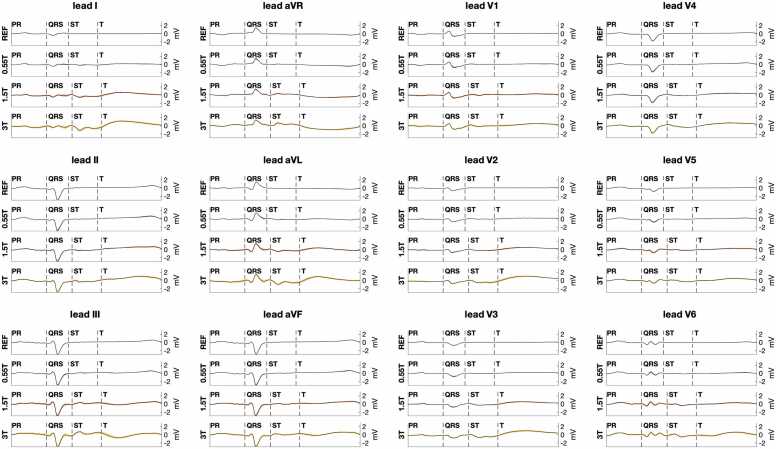
Illustration of reduced ECG variation after cinching together ECG lead wires. The top trace for each lead is the baseline ECG outside the scanner room (labeled REF). ECGs at MRI scanner isocenter are shown for 0.55T (second trace), 1.5T (third trace), and 3T (fourth trace). The black line for each trace is the average ECG value for each heartbeat time point over a 2-minute ECG recording. The colored bounds indicated the one standard deviation bound for each ECG time point. Standard deviation bounds are narrower for this ECG, with cinching together ECG leads, compared to the ECG in Fig. 2, without cinching of ECG leads. ECG: electrocardiogram, MRI: magnetic resonance imaging.

**Fig. 7:**
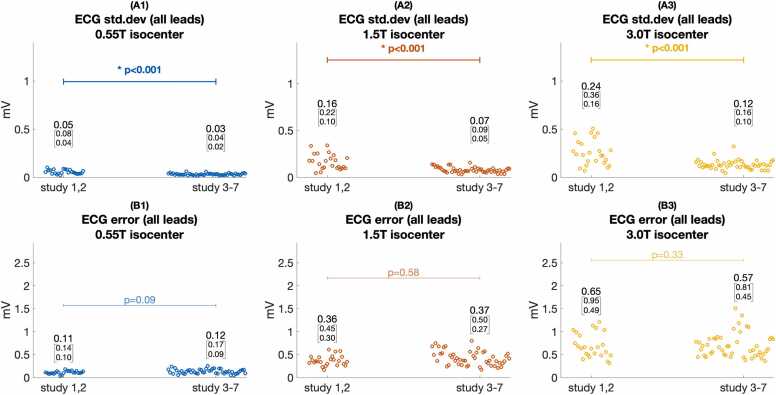
Effect of cinching together ECG lead wires on ECG distortion. (A1–3) ECG standard deviation measurements and (B1–3) ECG error measurements before lead wires were cinched together (study 1, 2) compared to after leads were cinched together (study 3–7). ECG variation improved after lead cinching for all MRI scanner field strengths. However, ECG error was not affected by lead cinching. Figure data are from porcine subjects. ECG: electrocardiogram, MRI: magnetic resonance imaging.

Quantitative assessment of ECG error (Fig. 4) and standard deviation (Fig. 5) supported these findings. Fig. 4A shows ECG error was significantly lower at 0.55T compared to 1.5T and 3T (median 0.14 mV at 0.55T isocenter vs. 0.41 mV at 1.5T and 0.81 mV at 3T isocenter, p < 0.001). ECG error was also reduced by moving from scanner isocenter to home position (median 0.14 mV at 0.55T isocenter vs. 0.03 mV at 0.55T home position, p < 0.001). Fig. 4B shows median ECG error was greater for the ST interval compared to the PR interval at 1.5T and 3.0T (median 0.40 mV ST error vs. 0.16 PR error at 1.5T, p = 0.004), as expected from the relative MHD effect from aortic flow during versus before ventricular systole. At 0.55T, however, greater error for the ST segment did not reach statistical significance after Bonferroni correction (0.14 mV ST error vs. 0.07 mV PR error, p = 0.026). Fig. 5A shows ECG standard deviation was significantly reduced for 0.55T compared to 1.5T and 3T at scanner isocenter (median 0.06 mV at 0.55T isocenter vs. 0.14 mV at 1.5T and 0.26 mV at 3T, p < 0.005). Fig. 5B shows that at a given scanner field strength, ECG variation was not statistically different between PR, QRS, and ST intervals. Figs. 6 and 7 illustrate Cinching ECG leads together to minimize spacing between the lead wires was helpful for reducing beat-to-beat ECG variation at all scanner field strengths (e.g., at 1.5T median standard deviation was 0.16 mV before lead cinching vs. 0.07 mV after lead cinching, p < 0.001). Lead cinching did not appear to affect ECG error (e.g., at 1.5T median error was 0.36 mV before lead cinching vs. 0.37 mV after lead cinching, p = 0.37). An illustration of lead placement with and without lead cinching is provided in Supplementary Fig. 1.

### 3.2 Pre-clinical assessment of ischemic ST segment changes in the MRI scanner

**Fig. 8:**
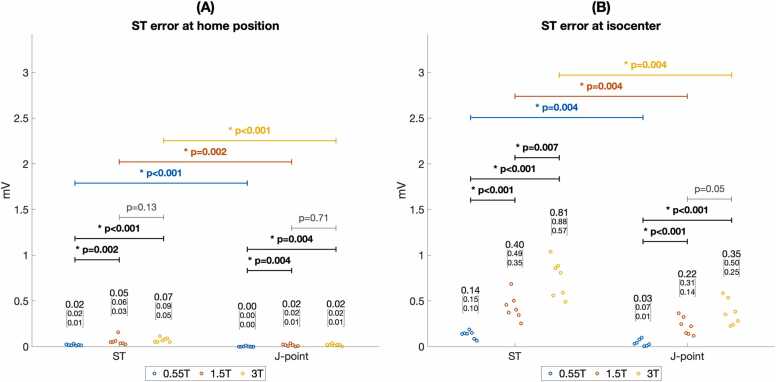
Error for detecting ischemic ST changes in 0.55T, 1.5T, and 3T MRI scanners. J point error, the primary time point used to detect ischemic ECG changes, was lower than overall ST error at all field strengths both at scanner table home position (A) and at isocenter (B). J point error and overall ST error were significantly lower at 0.55T compared to 1.5T and 3T scanners. Figure data are from porcine subjects. ECG: electrocardiogram, MRI: magnetic resonance imaging.

Fig. 8 shows ST segment error was significantly lower at 0.55T compared to 1.5T and 3T both at scanner isocenter (median 0.14 mV at 0.55T vs. 0.40 mV and 0.81 mV at 1.5T and 3T, p < 0.001) and at scanner home position (median 0.02 mV at 0.55T vs. 0.05 mV and 0.07 mV at 1.5T and 3T, p < 0.005). Only at home position in the 0.55T scanner could ST segment error fall within the strict 0.025 mV guideline recommended ceiling for ECG distortion [14].

The J point measurement, used for assessing myocardial ischemia and infarction, had significantly lower error than the error over the entire ST segment both at scanner isocenter (Fig. 8B) and at scanner home position (Fig. 8A). For example, at 1.5T the median error at isocenter was 0.40 mV for the ST segment vs. 0.22 mV for the J point, p = 0.004. The median error at the 1.5T scanner home position was 0.05 mV for the ST segment vs. 0.02 for the J point, p = 0.002. At isocenter, only in the 0.55T scanner was the J point error within the 0.1 mV threshold for assessing ischemic ST segment deviation (median 0.03 mV, IQR 0.01–0.07 mV) [16], [18].

**Fig. 9:**
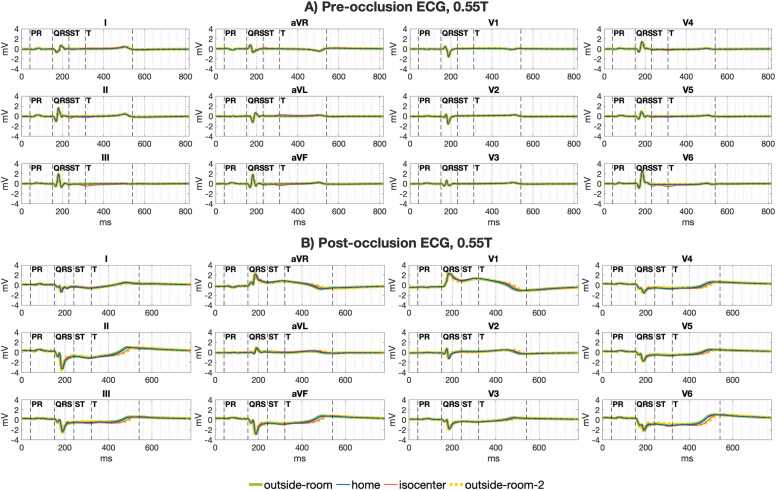
Illustration of ischemic ECG changes before and after porcine coronary artery occlusion in a 0.55T scanner. (A) Shows the baseline 12-lead ECG prior to coronary artery occlusion outside the MRI scanner (green line), at scanner home position (blue line), and at isocenter (red line). The ECG at scanner home position closely approximates the ECG outside the MRI. Some deviation of the ECG at scanner isocenter is noted within the ST segment and T wave intervals compared to the ECG outside the scanner. (B) Shows marked ST segment deviation is seen in several ECG leads after coronary artery occlusion. Changes in the QRS complex shape are also seen. Good agreement of gross ST segment and QRS changes is noted outside the scanner room (green line) compared to scanner home position (blue line) and scanner isocenter (red line). More subtle deviations between the ECG outside the scanner room and inside the MRI scanner are within the range noted for the repeat ECG outside the MRI scanner (dashed yellow line). ECG: electrocardiogram, MRI: magnetic resonance imaging.

**Fig. 10:**
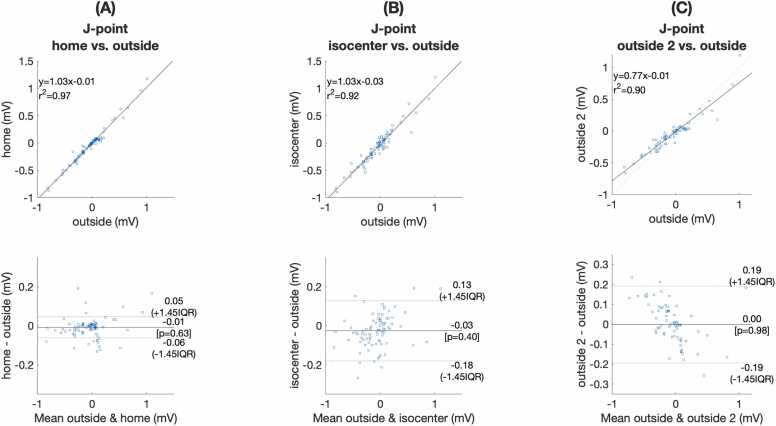
Correlation of ischemic ST assessment inside vs. outside the 0.55T MRI scanner following porcine coronary artery occlusion. J point measurements including all 12 leads from 7 subjects are shown. There was good correlation of the J point measurement outside the MRI scanner compared to the J point measurement (A) at scanner home position (r^2^ = 0.97) and (B) at isocenter (r^2^ = 0.92). J point correlation inside the scanner was within the range of (C) repeat measurements outside the scanner room (r^2^ = 0.90). J point changes between repeat ECGs outside the scanner room reflect true variations in the ST segment during coronary artery occlusion. ECG: electrocardiogram, MRI: magnetic resonance imaging.

Fig. 9 illustrates the ability to assess ST changes during coronary occlusion induced ischemia in a 0.55T scanner. Fig. 10 shows good correlation between the reference J point deviation outside the scanner room compared to J point deviation at scanner home position (r^2^ = 0.97, Fig. 10A) and scanner isocenter (r^2^ = 0.92, Fig. 10B). This correlation was within that observed for repeat ECG recording made outside the scanner room (r^2^ = 0.90, Fig. 10C). Diagnostic assessment of ischemic ECG changes inside the scanner agreed with ischemia assessment outside the scanner for 13 of 14 ECGs (92%). The one case of disagreement was due to ischemia being overcalled inside the scanner because of MHD related ECG distortion close to the J point (Supplementary Fig. 2). Supplementary Fig. 3 illustrates the potential for detecting ST elevations on the 12-lead ECG at 0.55T isocenter prior to averaging. The ability to detect and characterize an isolated PVC at 0.55T isocenter without averaging is also illustrated on this tracing.

### 3.3 Assessment of resting ST segment distortion in human subjects at 0.55T

**Fig. 11:**
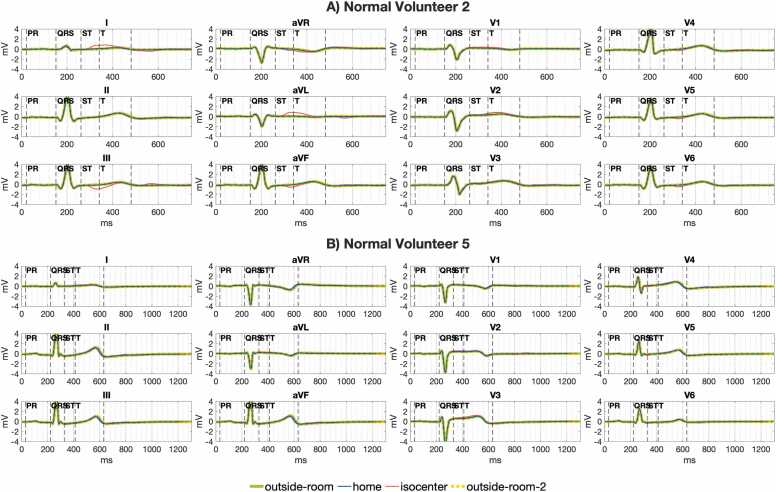
Illustration of ST interpretation for human volunteers in a 0.55T MRI scanner. (A) A volunteer with ST segment and T wave distortion at scanner isocenter (red ECG trace) compared to outside the scanner room (green ECG trace). Though the ST segment is distorted in several leads, interpretation of ST segment deviation at the J point (at the start of the ST segment) appears preserved. (B) A volunteer with preserved ECG morphology at scanner isocenter compared to outside the MRI (overlapping green and red ECG traces). ECG: electrocardiogram, MRI: magnetic resonance imaging.

ST segment distortion was greater in human volunteers than for the animal study both at 0.55T scanner isocenter (median error 0.35 mV volunteers vs. 0.14 mV animals, p = 0.005) and at scanner home position (median error 0.04 mV volunteers vs. 0.02 mV animals, p = 0.03). The increased ST distortion in volunteers was due to greater ECG error (Supplementary Fig. 4A1) and not greater ECG variation (Supplementary Fig. 4A2). This suggest larger MHD effect between the volunteers and animals, likely due to differences in lead spacing with larger torso size and potentially from differences in aortic flow relative to lead orientation. Supplementary Fig. 4B shows for volunteers, as with animals, J point error was significantly less than error over the entire ST segment. For example, at 0.55T isocenter the median error for volunteers was 0.06 mV for the J point vs. 0.35 mV for the ST segment, p = 0.16. At 0.55T home position, the median volunteer J point error was below the 0.025 mV guideline recommended ceiling for ECG distortion (median error 0.00 mV, IQR 0.00–0.01 mV). At 0.55T scanner isocenter, the median volunteer J point error was below the 0.1 mV guideline threshold for detecting cardiac ischemia (median error 0.06 mV, IQR 0.05–0.12 mV). Fig. 11 illustrates 12-lead ECGs in human volunteers with greater and smaller levels of ST segment distortion at 0.55T scanner isocenter.

### 3.4 Changes in ST segment distortion with ergometer stress in human subjects at 0.55T

Following ergometer stress, ECG distortion encroaches closer to the J point compared to the resting ECG at when the subject is at scanner isocenter (Supplementary Fig. 5). This illustrates that increased cardiac output during stress testing and associated increase in MHD may further limit assessment of J point deviation compared to ECG monitoring applications that do not increase cardiac output. At home position, ST segment and J point distortion were not noted at rest or following ergometer stress. This supports the feasibility of stress ECG interpretation at 0.55T home position.

### 3.5 Relationship of aortic flow to ST segment distortion at 0.55T

We found that the greatest aortic flow by imaging occurred 114 ms after the J point [IQR 84–115 ms]. Maximum ECG distortion across all leads occurred 29 ms after peak aortic flow [IQR −7 ms to +91 ms]. These findings support the relative preservation of the J point assessment compared to later portions of the ST segment and T wave. Supplementary Fig. 6 illustrates the relationship aortic flow to ST segment distortion in a subject with prominent ST segment distortion. Placement of leads for the 12-lead ECG recordings did not appear to affect image quality during aortic flow localizers and imaging.

### 3.6 Comparison of error between leads of the 12-lead ECG

This study appeared underpowered to detect differences in distortion between ECG leads. In the 3T scanner, contiguous lead error for lateral leads (I, aVL, V5, and V6) was greater than the error for septal (V1 and V2) and anterior (V3 and V4) precordial leads (Supplementary Fig. 7A). This is consistent with the expectation that leads perpendicular to blood flow in the aortic arch, such as lead I, are most affected by MHD induced voltage [4]. However, this pattern was not detected in the 1.5T or 0.55T scanners (Supplementary Fig. 7B, C) or between individual leads in the 3T scanner (Supplementary Fig. 8).

## 4 Discussion

This study evaluated 12-lead ECG interpretation in a low field 0.55T MRI scanner. ECG distortion in the MRI environment remains a barrier for applying MRI to procedures where ECG detection of cardiac ischemia and arrhythmia is part of standard clinical practice, such as cardiac interventional procedures and cardiac stress testing. ECG distortion is known to decrease with decreasing MRI field strength. This study characterizes the advantages and limitations of ECG interpretation in a 0.55T MRI scanner.

MRI ECG distortion can be separated into distortion related to MHD, and distortion due to magnetically induced currents from patient movement and from MRI scanning [23]. We found that MHD-related ECG distortion, mostly involving the ST segment, was significantly improved at 0.55T compared to 1.5T and 3.0T. We found motion-related ECG distortion was also significantly improved at 0.55T compared to 1.5T and 3.0T, was largely due to respiratory motion and was manifest as beat-to-beat variability that affected all ECG segments. This study did not evaluate ECG distortion during MRI scanning, which is discussed further below.

The guideline suggested 0.025 mV ceiling for ECG distortion appears achievable at scanner home position at 0.55T. This may simplify acquisition of diagnostic quality ECG shortly after stress testing or during MRI-guided cardiac interventions, avoiding more cumbersome patient repositioning needed for accurate ECG acquisition around higher field scanners [8]. ECG interpretation at scanner isocenter is discussed further below.

### 4.1 12-Lead ECG interpretation of myocardial ischemia inside the MRI scanner

Though MHD distortion of the ST segment has generally be considered to preclude ECG-based assessment of myocardial ischemia at 1.5T and 3T, some interpretation of ischemic ECG changes appears possible inside a 0.55T scanner bore. Diagnosing ventricular ischemia and infarction on ECG are based primarily on the deviation of the J point at the end of the QRS complex from the ECG baseline between the T wave and subsequent QRS complex [18]. J point deviations of 0.1 mV or greater in two contiguous ECG leads are considered significant [24]. At 0.55T isocenter, the median resting J point error was below the 0.1 mV threshold for assessing ischemia (0.03 mV in animals and 0.06 mV in volunteers). Though this error can obscure subtle signs of ischemia, we found that clinician diagnosis of ischemic ECG changes during experimental coronary artery occlusion is possible. J point error was significantly higher at 1.5T and 3T (median 0.24 mV and 0.42 mV, respectively) supporting consensus that ST segment interpretation is currently not possible inside MRI scanners with higher field strength. When cardiac output is increased, as during stress testing, J point distortion may increase and further obscure signs of ischemia inside the MRI scanner but did not appear to affect ST segment interpretation at the 0.55T scanner table home position.

The shape of the ST segment from the J point to 80 ms after is secondarily used for differentiating ischemic from non-ischemic J point deviation [9]. We found overall ST segment error was higher than J point error at all field strengths. This limits interpretation of ST shape characteristics such as down-sloping versus up-sloping ST depression inside the scanner. At 0.55T, observation of concerning J point deviation might trigger moving the subject to scanner home position to assess ST segment changes more completely.

Additional mitigation of ST segment distortion may be possible. Leads oriented parallel to the MHD voltage are expected to have higher MHD distortion. Kakareka et al. suggested passing lead cables to one side of the aorta to reduce the contribution of cable routing on this effect [25]. Conventional filtering is limited at suppressing MHD distortion because its frequency spectrum and magnitude overlap that of the ECG signal. Other filtering approaches are being investigated. Tse et al. used adaptive filtering to estimate the MHD voltage using a reference ECG taken outside the scanner [26]. The MHD voltage could then be subtracted from the MRI ECG to estimate the signal without MHD distortion. Separate filters were required for heartbeats that produce different cardiac outputs, such as PVCs. Bayesian filtering approaches are also being evaluated to address limitations of independent component analysis and Wiener filtering techniques [27], [28], [29]. The lower level of MHD noted at 0.55T compared to higher field MRI scanners may be more amenable to adequate suppression by these signal processing methods.

### 4.2 12-Lead ECG interpretation of cardiac arrhythmia inside the MRI scanner

Reduced ECG distortion aids interpretation of heart rhythm in lower field MRI scanners. As introduced in the Methods section, the P wave and QRS complex are the ECG features used for assessing heart rhythm. In contrast to MHD-related ECG distortion which affects the ST segment and T wave more than the P wave and QRS complex, respiratory motion associated beat-to-beat variability was noted to similarly affect the P wave, QRS complex, and ST segment and could have greater impact on rhythm interpretation. This study confirmed beat-to-beat variability was significantly lower at 0.55T compared to 1.5T and 3T. This translates to improved detectability of low amplitude features, such as the P wave, at 0.55T isocenter (Supplementary Fig. 1A1). By contrast, at 3.0T the P wave can fall within the range of beat-to-beat variation (Supplementary Fig. 1B3) and ECG distortion (Supplementary Fig. 1B5), making detection challenging. The shape of the higher amplitude QRS complex is better preserved between at 3.0T and 0.55T, though more distortion is noted in later portions of the QRS complex at higher field (Supplementary Fig. 1B5).

Several methods have been described to additionally reduce the effects of ECG beat-to-beat variability inside the MRI scanner and aid heart rhythm assessment. We found minimizing spacing between ECG leads by cinching them together and fixing ECG leads to the body with tape was useful for reducing respiratory motion-related ECG variability. Weighting ECG leads has also been used to reduce lead motion-related ECG distortion [26]. Beat-to-beat variability can be further suppressed by averaging, as performed in this study to isolate ECG error due to MHD effects. However, greater beat-to-beat variability at higher MRI field-strength takes longer periods of averaging to suppress. Averaging also reduces the ability to detect arrhythmia features that vary beat-to-beat such as the variable PR interval during higher-risk AV block or irregular fibrillatory P waves during atrial fibrillation. Without averaging, we found P wave interpretation was possible at 0.55T but challenging at higher field (e.g., in Fig. 1 and Supplementary Fig. 3). For assessment at higher field, irregular rhythms often have other ECG markers such as heart rate slowing or irregular durations between QRS complexes that could prompt moving to scanner home position if more accurate ECG interpretation is required. Intra-cardiac electrogram (ic-EG) recordings made by catheters that contact the atrium and ventricle can also accurately differentiate such irregular arrhythmias during invasive electrophysiology procedures [30]. ic-EG is typically acquired by bipolar measurement using intravascular catheters with closely spaced electrodes and wires that reduce sensitivity to remote EM fields. Though ic-EG is not used for ischemia assessment, they are suitable for heart rhythm assessment at 1.5T and potentially higher field scanners [3], [31], [32].

At 0.55T, P wave and QRS distortion after averaging did not appear to limit interpretation and were less sensitive to lead placement. At higher field strength, potentially limiting ECG errors in the P wave and toward the end of the QRS complex were observed even after averaging (e.g., in Figs. 1 and 2). Oscillations in the ECG baseline that obscure the P wave could be due to insufficient averaging in the 2-minute recordings and MHD effects in cases where the T wave from one heartbeat encroaches on the P wave from the next heartbeat. ECG error toward the end of the QRS complex is consistent with the delay in electro-mechanical coupling during QRS complex and resulting systolic MHD affecting later parts of the QRS more than initial portions of the QRS. Such QRS distortion might reduce the accuracy of localizing ventricular conduction abnormalities or ectopic ventricular rhythms at higher MRI field-strength. This distortion appeared less prominent after minimizing spacing between ECG leads (Fig. 5).

### 4.3 Differences in 12-lead ECG systems from conventional MRI ECG

Conventional MRI ECG system design has focused on accurate QRS complex detection to synchronize imaging to the cardiac cycle and reducing the risk of lead heating which can lead to skin burns [7]. However, several design optimizations limit the diagnostic utility of MRI ECG systems compared to clinical 12-lead ECG. ECG lead coupling with the RF transmit field can cause lead heating and coupling with the position encoding gradient fields can cause prohibitive ECG noise. Shorter leads and fewer leads are generally used for MRI ECG to reduce coupling with these MRI generated electromagnetic (EM) fields [33], [34]. MRI ECG leads are also typically placed in non-standard locations chosen to minimize MHD [35]. Fewer leads with non-standard location limit the ability to anatomically localize ECG findings compared to 12-lead ECG [18], [36]. MRI ECG also often uses high impedance leads to attenuate induced currents from MRI EM fields [7], [37]. However, high lead impedance also reduces ECG signal amplitude and increases noise [26]. Finally, while clinical ECG filtering has been standardized to ∼0.5–150 Hz, more restrictive filtering is generally used in MRI ECG systems since most of the QRS complex spectrum is in the 13–21 Hz band [38]. Higher low-frequency cutoffs above 1 Hz can help improve QRS complex detection by suppressing lower frequency MHD and respiratory motion effects [39]. However, this increases distortion of the ST segment and T waves, which are important for diagnosis of cardiac ischemia [21]. Lower high-frequency cutoff around 20 Hz can suppress EM interference (EMI) from the gradient fields [40]. However, this alters the amplitude and shape of the QRS complex which restricts clinical interpretation [21].

Interest in performing interventional procedures under MRI guidance has renewed interest in improving the diagnostic quality of ECG in the MRI scanner. ECG distortion due to the static magnetic field is considered a major factor limiting ECG interpretation in higher field MRI systems, such as 1.5T and 3.0T. This work focused on better understanding the degree of improvement in ECG interpretation afforded by a lower field 0.55T system. However, this work only reflects ECG interpretation that is possible between MRI scans and when the subject is at scanner home position. During MRI scanning, gradient EMI additionally limits ECG interpretation in both low-field and higher-field MRI scanners. Tse and colleagues proposed several modifications to 12-lead ECGs systems for improved compatibility with the MRI while preserving diagnostic utility [26]. The 12-lead ECG uses longer leads than typical MRI ECG systems. To avoid ECG signal attenuation from long, high impedance ECG leads, they used low impedance coaxial cable. Lead heating from RF induced currents was instead suppressed using ferrite chokes. For suppressing gradient EMI, their group proposed high bandwidth (45 kHz) and amplitude ECG acquisition to avoid distortion of the gradient EMI, establishing a transfer function between the gradient control signals (GCS) and resulting EMI using ECG during and between MRI scans, and using this transfer function and the GCS to estimate and subtract this EMI from the ECG signal [41]. Adaptive filtering utilizing the GCS as input has also been used to suppress gradient EMI, and is being evaluated for MRI conditional patient ECG monitoring [25], [40]. Commercial development of MRI-conditional 12-lead ECG systems that incorporate gradient EMI suppression is underway but remains a work-in-progress (PinMed, Pittsburgh, Pennsylvania, USA; MiRTLE Medical, North Andover, Massachusetts, USA).

### 4.4 Other considerations for performing interventional MRI procedures at 0.55T

Apart from improved ECG interpretation, other potential benefits of using low-field MRI for performing interventional MRI procedures include reduced SAR, which improves heating safety of interventional devices, less restrictive scanner bore size and geometry, and reduced gradient noise [11], [13]. Low field MRI systems also have lower installation and maintenance cost and reduced sensitivity to magnetic susceptibility artifacts [13]. The latter improves MRI assessment of the lungs and may have application to imaging near metallic implants, such as pacemakers and implantable defibrillators [11].

However, whether these benefits of low-field MRI systems outweigh restrictions on temporal and spatial resolution compared to higher field systems still needs to be determined. Image SNR is proportional to field strength and reduced SNR is a major limitation of lower-field MRI systems. Work is ongoing into methods for optimizing SNR efficiency taking advantage of the shorter T1 at low field which permits shorter TR imaging, and longer T2 and T2* which permit longer read-outs [11]. Utilizing such methods, modern low-field MRI systems are capable of technically demanding imaging, such as cardiac and real-time MRI [42]. Machine learning-based image reconstruction may further mitigate SNR restrictions of low field MRI systems [43], [44].

The optimal field strength for balancing image SNR and ECG interpretability remains an open question. Jekic et al. found that accurate ECG can be acquired at an MRI scanner fringe field of 70 mT [8]. Our study utilized a 0.55T system as scanners with this field strength are commercially available, are capable of cardiac imaging, and are being evaluated for interventional MRI applications where ECG monitoring is desirable. We found that clinical ECG interpretation appears feasible at the scanner table home position and that important features, such as J point deviation, have some interpretability at the 0.55T scanner isocenter. Though ECG interpretability is expected to further improve at lower field than 0.55T, we anticipate that the tradeoff of lower SNR will make such systems less suitable for interventional MRI applications.

### 4.5 Limitations

Several limitations of this study are acknowledged. Small sample size limited detection of differences in ECG error and variation between individual leads. For statistical comparison, we instead used the highest error and variation among all 12 leads to reflect the lead that could most compromise ECG interpretation. The increased ECG distortion in volunteers compared to animals suggests an effect of torso size. More human subjects, including larger subjects, are needed to better characterize expected ECG distortion in the clinical setting. The method for determining error between ECG measurements has not been standardized and error results could vary for different methods [7]. We applied a method of error measurement that optimized alignment of the P wave and QRS complex which were expected to be less affected by MHD distortion. The resulting ECG alignment corresponded with visual assessment of optimized alignment. A prior study of ST segment error in a 1.5T MRI scanner used an ST segment duration of 120 ms following the J point compared to 80 ms in this study [8]. We chose 80 ms as a clinically suggested duration for assessing ischemic ST segment changes and found that increasing the ST segment to 120 ms did not change study conclusions. This study used long 2-minute recordings so that averaging of individual heartbeats could better distinguish cardiac cycle synchronized ECG distortion, such as MHD effects, from non-cardiac sources such as respiratory motion. Shorter periods of averaging or no averaging may be useful for ECG monitoring. The effect of less averaging on ECG error requires further study. We chose to exclude the T wave when presenting ECG error and variation results in Figs. 2 and 3. When including the T wave, the median ECG error at home position was unexpectedly high for the 1.5T scanner compared to the 3T scanner (Supplementary Fig. 9A1). ECG error at home position followed the expected increase with increasing field strength when the T wave was excluded (Supplementary Fig. 9A2). Including the T wave did not affect conclusions related to ECG variation (Supplementary Fig. 9B1,2). The greater-than-expected ECG error at 1.5T home position was caused by time shifting of the T wave compared to the reference ECG outside the scanner room rather than MHD-related T wave distortion. This was likely due to isoflurane anesthesia effects on cardiac repolarization when isoflurane was restarted following transport of animals from the 0.55T scanner area to the 1.5T scanner [45]. Allowing more time to reach a stable QT interval and heart rate after initiating anesthesia could address this issue in future studies. During the coronary occlusion study, changes in ST deviation were noted between repeat ECG recording outside the scanner room. We attempted to minimize ST segment changes between ECG recordings by waiting 10–15 min for qualitative stabilization of ST deviation before the first recording. A quantitative measurement of ST deviation may have prompted waiting for a higher degree of ST segment stability and improved the correlation between reference ECG measurements outside the scanner room. This could allow more precise evaluation of detecting ischemic ST segment changes inside the MRI scanner. During the ergometer stress study, assessment of ST segment distortion between scanner table home position and isocenter was qualitative rather than quantitative. Quantitative analysis was confounded by increased ECG baseline drift due to increased respiratory motion after exercise, variations in the peak stress heart rate at home position versus isocenter, and some observed changes in QRS duration which shifted the location of the J point between rest and stress ECGs. Baseline drift removal methods were noted to alter the ST segment morphology and longer averaging to averaging to suppress baseline drift was not used because of the rapid decline in heart rate after stopping exercise. Dobutamine stress assessment was not available for human subjects at our site but would have avoided the issue of exercise increases in respiratory motion and permitted maintenance of higher, more uniform heart rates during stress ECG recordings.

## 5 Conclusions

ECG distortion is significantly improved in 0.55T compared to 1.5T and 3T MRI scanners due to reduced MHD effects and reduced sensitivity to subject motion, such as respiration. At scanner home position, ECG distortion at 0.55T is low enough that clinical interpretation appears feasible without need for more cumbersome patient repositioning. At scanner isocenter, interpretation of P waves and the QRS complex appears sufficient for distinguishing most clinically relevant arrhythmias, particularly if averaging across multiple heartbeats can be used. Marked ST segment changes, such as ST elevation during coronary occlusion, also appear detectable at 0.55T isocenter. However, the level of distortion remains enough to obscure subtle ST segment changes that could be clinically relevant. The reduced level of ECG distortion in 0.55T scanners may simplify the problem of suppressing residual distortion by ECG cable positioning, averaging, and filtering and could reduce current restrictions on ECG monitoring during interventional MRI procedures.

## Funding

This work was supported by the Division of Intramural Research, 10.13039/100000050National Heart, Lung, and Blood Institute, 10.13039/100000002National Institutes of Health, Bethesda, Maryland, USA (Z01‐HL005062, Z01‐HL006061, Z1A‐HL006213, and ZIA-HL006257).

## Author contributions

**Daniel A. Herzka:** Writing – review and editing, Writing – original draft, Visualization, Validation, Methodology, Investigation, Formal analysis, Data curation, Conceptualization. **Robert J. Lederman:** Writing – review and editing, Supervision, Resources, Project administration, Funding acquisition, Conceptualization. **Adrienne E. Campbell-Washburn:** Writing – review and editing, Resources, Investigation. **Dursun Korel Yildirim:** Writing – review and editing, Methodology, Investigation, Conceptualization. **Felicia Seeman:** Writing – review and editing, Investigation. **Christopher G. Bruce:** Writing – review and editing, Investigation. **Aravindan Kolandaivelu:** Writing – review and editing, Writing – original draft, Visualization, Validation, Software, Project administration, Methodology, Investigation, Formal analysis, Data curation, Conceptualization.

## Ethics approval and consent

Animal protocols were reviewed and approved by the Animal Care and Use Committee at the National Institutes of Health. The healthy volunteer protocol was approved by the Institutional Review Board of the National Institutes of Health (ClinicalTrails.gov identifier NCT03331380), and all subjects provided written informed consent.

## Consent for publication

Consent for publication was obtained for the healthy volunteer ECGs presented in Fig. 11 and Supplementary Figs. 5 and 6.

## Declaration of competing interest

The authors declare that they have no known competing financial interests or personal relationships that could have appeared to influence the work reported in this paper.

## Data Availability

The datasets generated during and/or analyzed during the current study are available from the corresponding author on reasonable request.
