# Supplementary material for: Evaluation of 12-lead electrocardiogram at 0.55T for improved cardiac monitoring in magnetic resonance imaging
Source: J Cardiovasc Magn Reson. 2024 Feb 10;26(1):101009. doi: 10.1016/j.jocmr.2024.101009 (PMC10940178; doi:10.1016/j.jocmr.2024.101009)
Supplement: Supplementary file 1 — Supplementary material [file mmc1.docx]

**Supplementary Figure 1:**

Illustration of ECG lead placement A) without attention to minimizing spacing between lead wires vs. B) Cinching leads together to minimize spacing between lead wires.


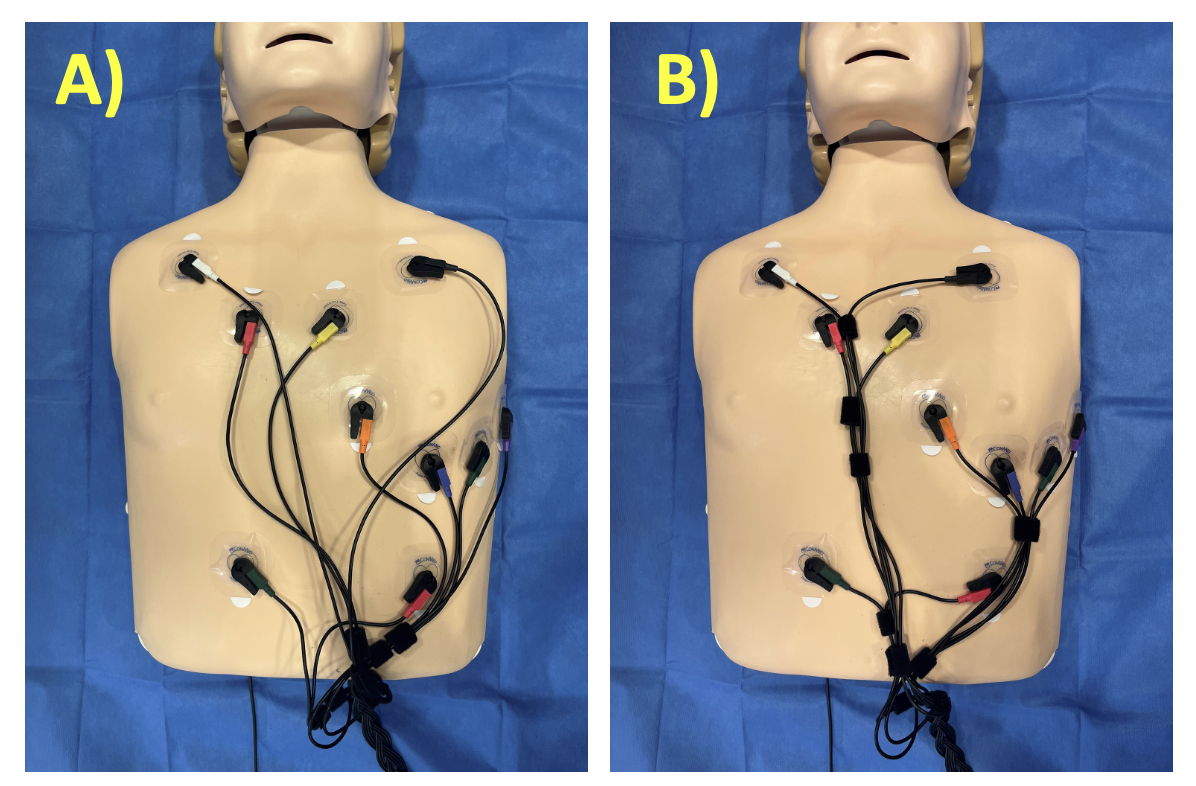


**Supplementary Figure 2:**  Case of incorrectly classified ischemic ST deviation in a 0.55T MRI scanner. During reader ECG interpretation, ECG traces outside vs. inside the scanner were presented individually rather than overlayed as shown in this plot. Outside the scanner room there is low suspicion for ischemic J-point deviation (green ECG trace). The ECG at scanner isocenter (red trace) was misclassified as having ischemic J-point deviation due to MHD related ECG distortion close to end of the QRS complex (for example in lead V5 and V6). Registering the reference ECG outside the scanner room to in-scanner ECGs, as shown in this plot, may help determine the correct end of the QRS complex and reduce misclassification of J-point deviation.

**
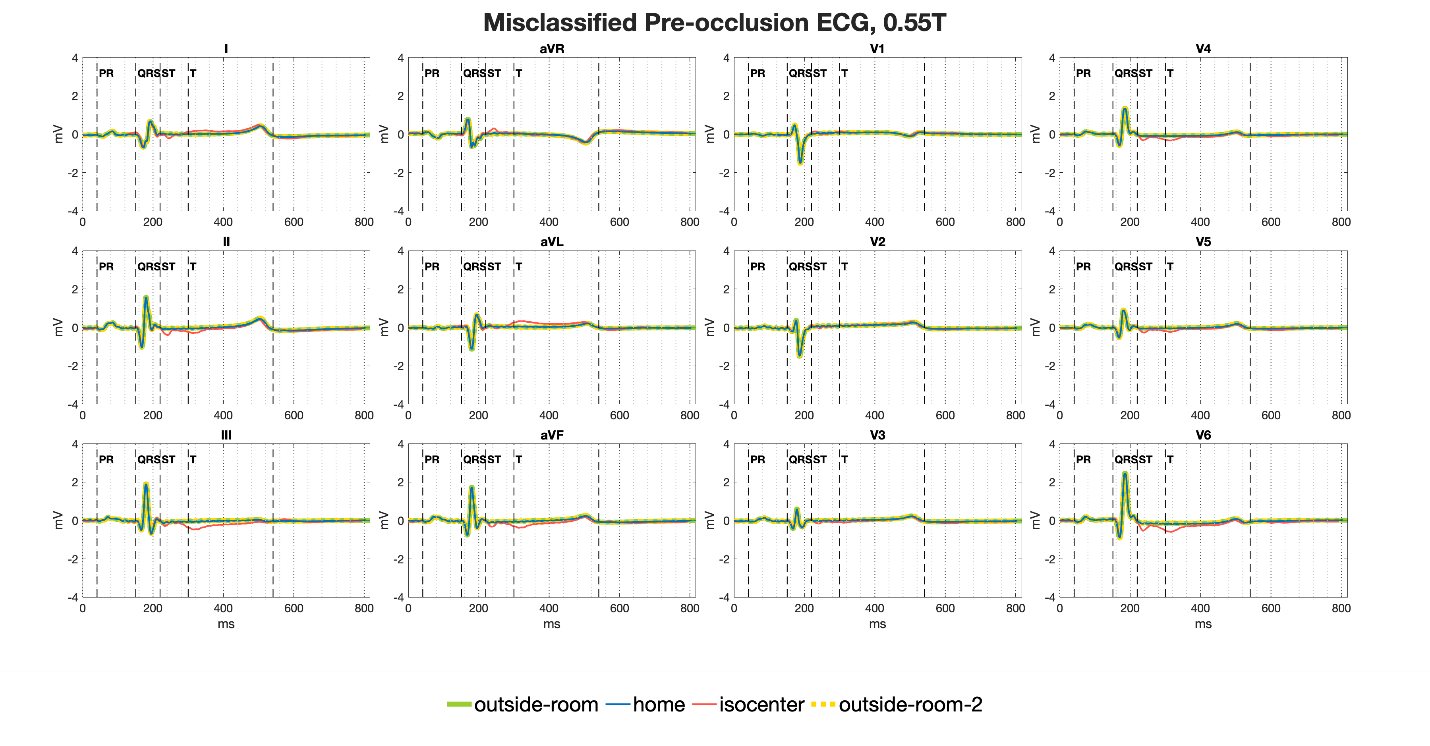
**

**Supplementary Figure 3:** Detection of arrhythmia and ischemic ST segment changes on the 12-lead ECG at 0.55T isocenter prior to averaging. Red arrows highlight a premature ventricular complex (PVC), which appears to have a wider-QRS complex and absence of preceding P wave. The PVC axis (positive QRS deflection in V1, negative QRS in V2-V6, negative in lead I, and negative in II,III, and aVF) suggests PVC origin from a left ventricular inferior apical focus. The blue arrow highlights ST elevation in lead V1 that can be interpreted without averaging.


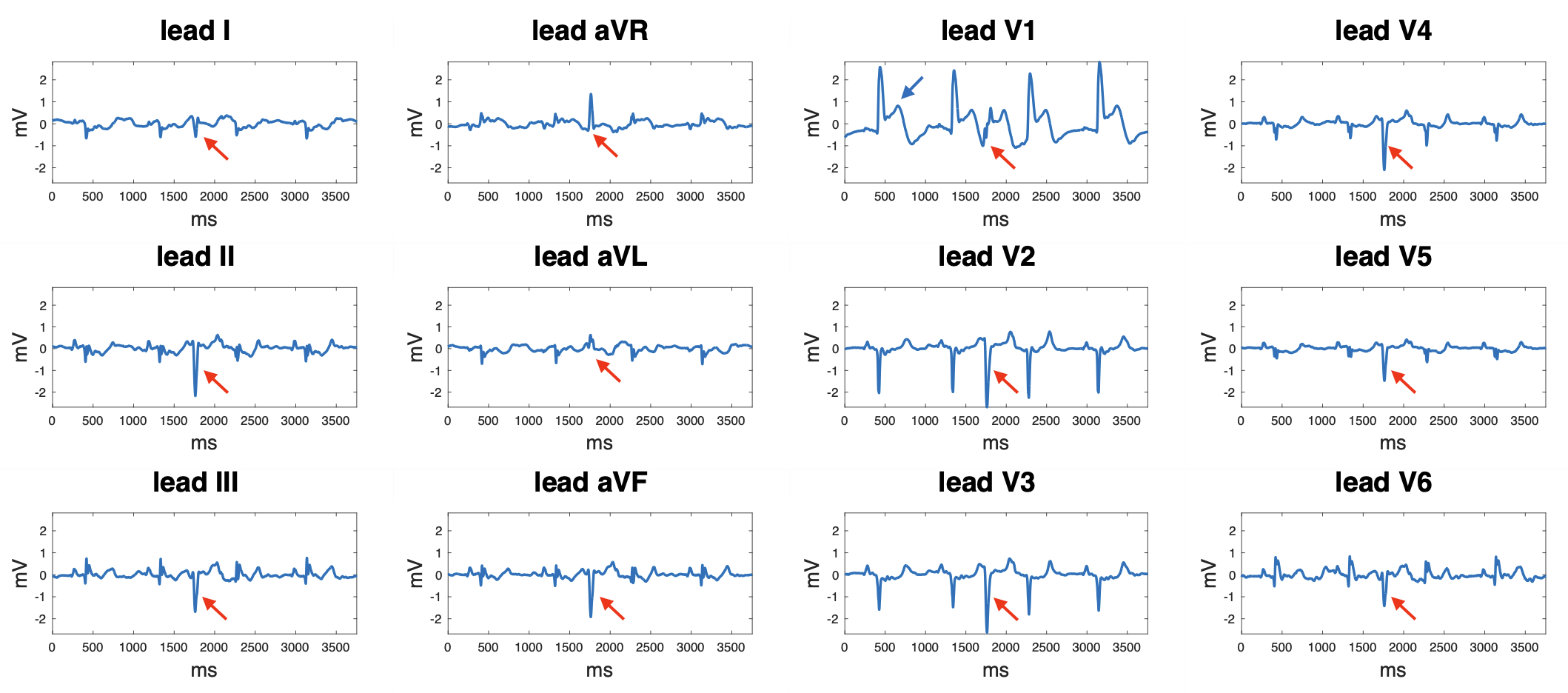


**Supplementary Figure 4:** ST segment distortion in porcine subjects compared to human volunteers in a 0.55T scanner. A1-2) Overall ST segment error was higher for human subjects both at scanner home position and at isocenter. ST segment variation was not significantly different between human subjects and volunteers. These findings are consistent with greater ECG distortion in human volunteers being due to greater MHD effect rather than subject motion. B1-2) J point error was significantly lower than overall ST segment error for both porcine and human subjects at scanner home position and at isocenter.


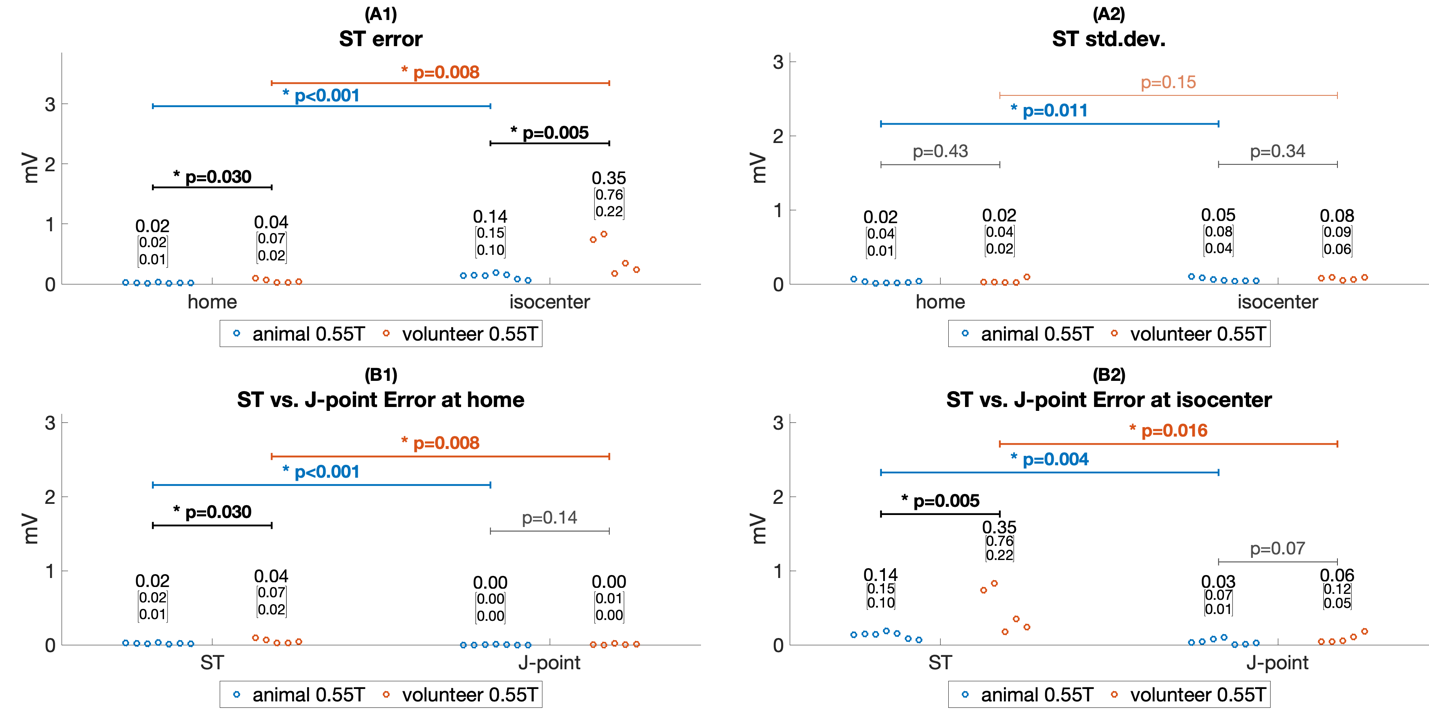


**Supplementary Figure 5:** Example of ST segment distortion during ergometer stress in a human subject at 0.55T. At scanner table home position, ST segment distortion is not noted at rest (light blue ECG tracing) or after stress (dark blue tracing). At isocenter, distortion of the ST segment and T wave are noted at rest (dashed light red ECG tracing) and distortion increases with stress (solid red tracing). At rest, distortion is more apparent later in the ST segment and the J point immediately following the QRS complex does not appear grossly distorted. With stress, distortion is apparent earlier in the ST segment and encroaches on the J point. In this example, some distortion of the J point is observed in leads I, aVL, II, III, and aVF.

**
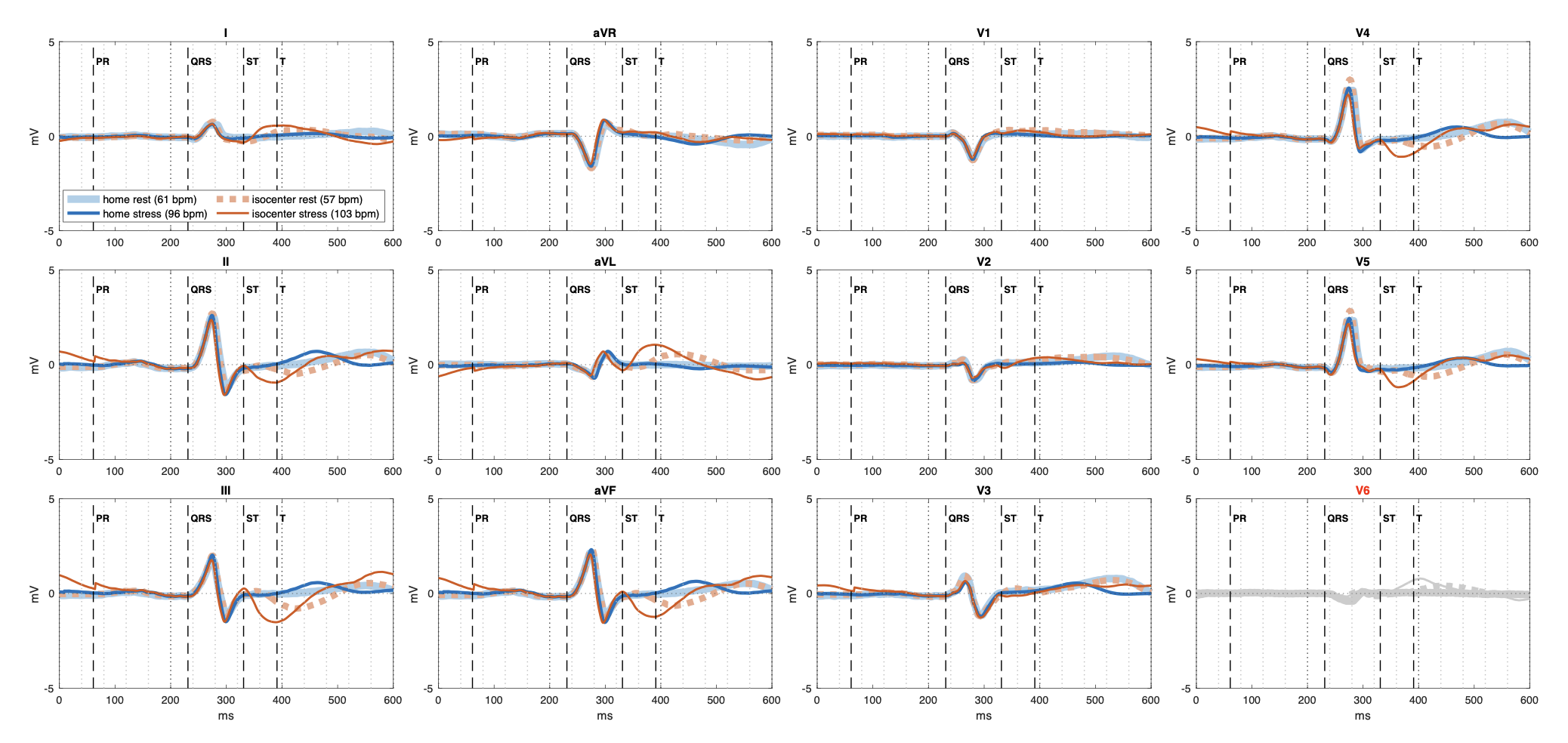
**

**Supplementary Figure 6:** Relationship of aortic flow to ST segment distortion at 0.55T isocenter. Aortic flow by phase contrast MRI (red line) starts > 40ms after the beginning of the QRS complex and reaches a maximum toward the end of the ST segment/beginning of the T wave. ECG distortion (dashed purple line) is apparent during the ST segment, greatest during the T wave, and across subjects reached a maximum 29ms [IQR -7ms to +91ms] after peak aortic flow. ECG distortion was estimated as the absolute difference between the averaged ECG at scanner isocenter (dashed dark blue line) from the averaged ECG at scanner table home position (solid light blue line).


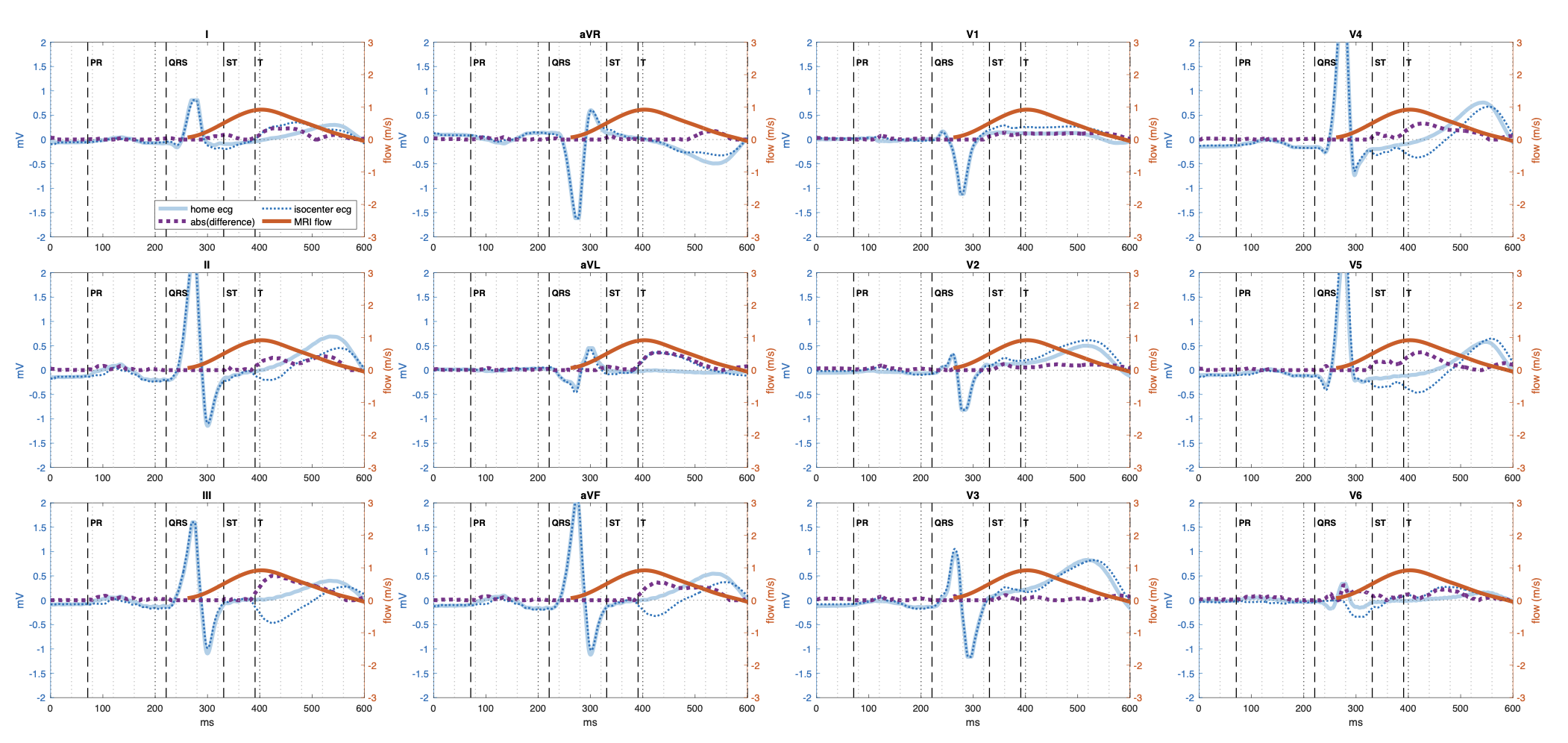


**Supplementary Figure 7:** ECG lead error by anatomic location. (A) in the 3T scanner, greater error was noted for the lateral leads compared to septal and anterior leads. However, this pattern was not detected for the (B) 1.5T or (C) 0.55T scanners possibly due to smaller level of error and insufficient sample size.


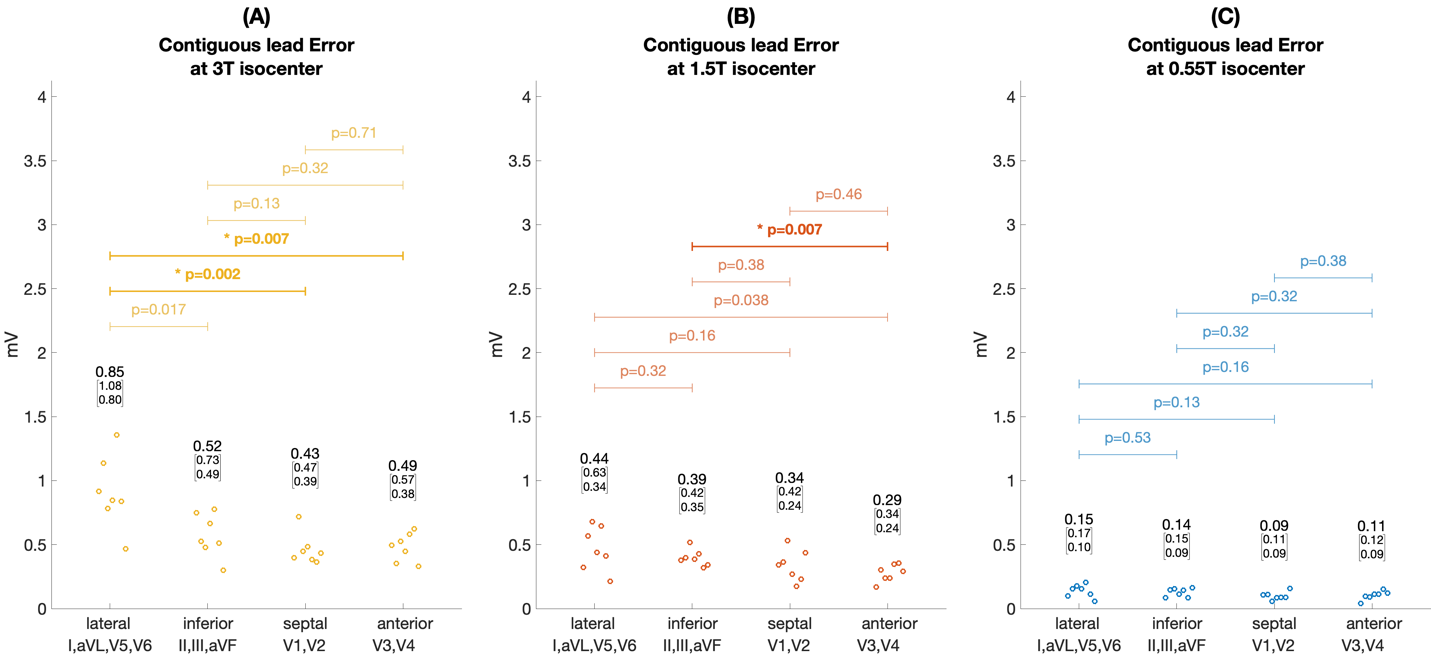


**Supplementary Figure 8:** Differences in error between individual leads of the 12-lead ECG in a 3T MRI scanner. No significant differences in error between ECG leads were noted after Bonferroni correction.


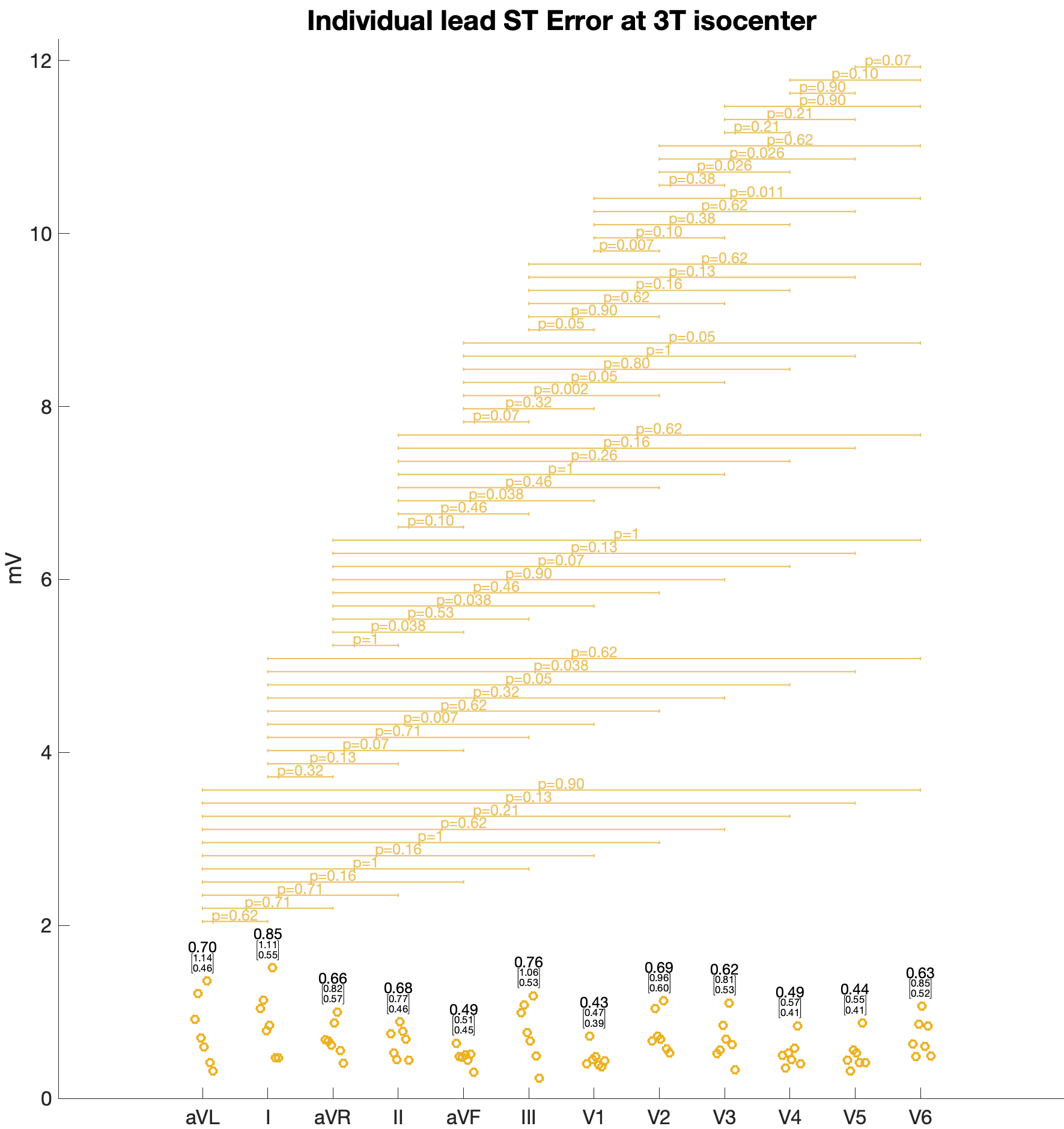


**Supplementary Figure 9:** ECG distortion in 0.55T, 1.5T, and 3T MRI scanners, including the T wave. A1) ECG error increased when moving from scanner table home position to isocenter in 0.55T and 3T scanners but did not reach statistical significance in the 1.5T scanner due to greater error at scanner table home position. A2) Home position error was significantly less when the T wave was excluded for the 1.5T scanner but not for other scanners. This was due to shifting of the T wave likely caused by restarting isoflurane anesthetic after transport to the 1.5T scanner versus more stable level of anesthesia for the other scanners. B1,2) Conclusions related to ECG standard deviation did not significantly change whether the T wave was included or excluded.

**
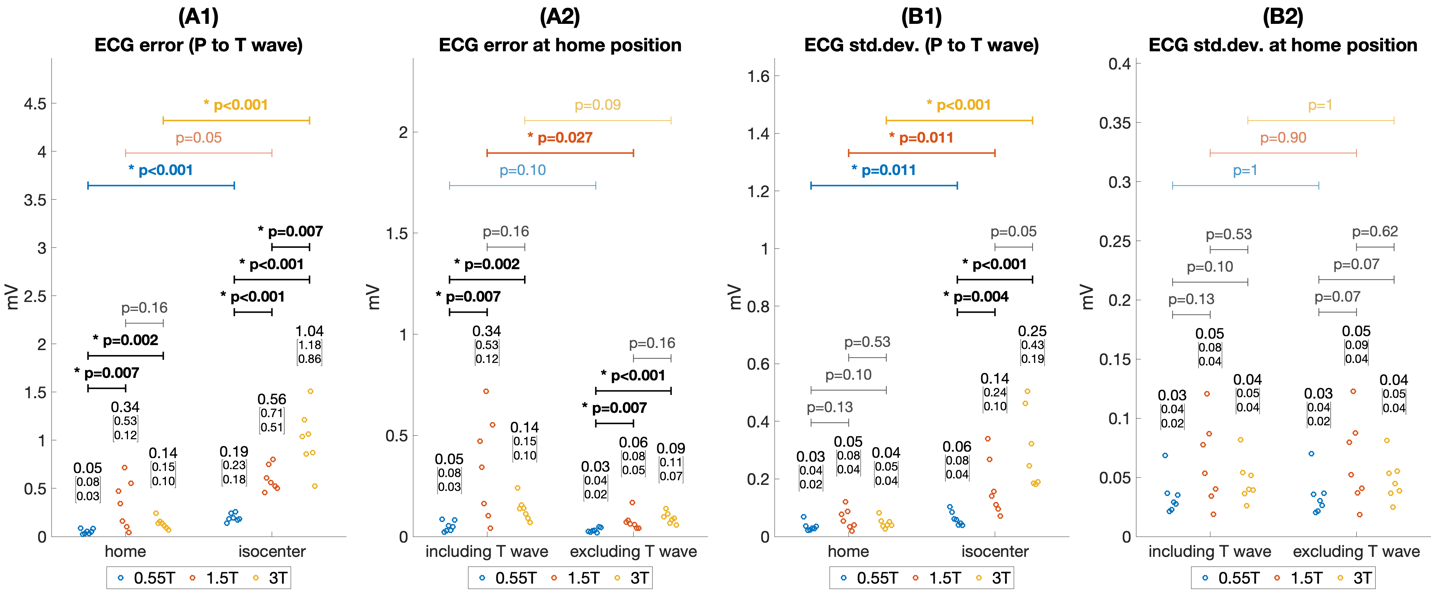
**
